# Renal Osteodystrophy as a Risk Factor for Postoperative Complications after Knee Arthroplasty: A National In-Patient Sample Study

**DOI:** 10.1055/a-2756-0149

**Published:** 2025-12-30

**Authors:** WeiLong Zhong, Binbin Zhu, Ying Xu, Hao Xie, ZhiGang Ai, Jian Wang

**Affiliations:** 1 Department of Anesthesiology, The Third Affiliated Hospital of Sun Yat-Sen University, Guangzhou, Guangdong, China; 2 Comprehensive Intensive Care Unit of The Third Affiliated Hospital of Sun Yat-Sen University, Guangzhou, Guangdong, China; 3 Division of Orthopaedic Surgery, Department of Orthopaedics, Nanfang Hospital, Southern Medical University, Guangzhou, Guangdong, China

**Keywords:** knee arthroplasty, renal osteodystrophy, complication, National Inpatient Sample

## Abstract

Renal osteodystrophy (ROD), a skeletal complication of chronic kidney disease (CKD)–mineral and bone disorder, may influence perioperative outcomes after total knee arthroplasty (TKA), but its impact remains unclear. This study examined patient characteristics, hospital resource utilization, and postoperative complications in ROD patients undergoing primary TKA. We performed a retrospective cohort analysis of the National Inpatient Sample (2010–2019). Adults undergoing primary TKA were identified and stratified by ROD status. Propensity score matching (PSM; 1:20) was used to balance age, sex, race, comorbidities, and CKD stage. Outcomes included length of stay (LOS), hospital charges, and medical and surgical complications. Among 1,196,522 TKA patients, 283 (0.02%) had ROD. After matching ( *n* = 5,337 controls), ROD patients had a longer median LOS (3 vs. 3 days; *p* < 0.001) and markedly higher median hospital charges ($58,550 vs. $18,004; *p* < 0.001). ROD was associated with increased odds of medical complications, including thrombocytopenia (OR: 3.2; 95% CI: 1.9–5.2), convulsion (OR: 6.9; 2.5–19.6), heart failure (OR: 2.3; 1.5–3.4), chest pain (OR: 3.4; 1.2–10.0), acute cerebrovascular disease (OR: 3.0; 1.4–6.4), stroke (OR: 3.3; 1.6–6.8), pneumonia (OR: 3.9; 1.7–9.0), and acute renal failure (OR: 2.3; 1.6–3.5). Surgical risks were also elevated, notably periprosthetic fracture (OR: 7.1; 2.2–22.9), joint dislocation (OR: 4.6; 1.7–12.3), and lower limb peripheral nerve injury (OR: 2.5; 1.4–4.7). ROD patients undergoing primary TKA incur greater hospital resource use and substantially higher rates of diverse medical and surgical complications. These findings highlight ROD as an independent risk factor warranting targeted preoperative risk stratification, multidisciplinary perioperative planning, and bone health optimization to improve outcomes and resource efficiency in this high-risk population.

The level of evidence is 3.

Trial registration is not applicable.

## Introduction

Total knee arthroplasty (TKA) is a surgical procedure that replaces a diseased knee joint with a prosthetic implant, aiming to restore normal joint function and relieve pain. It is widely recognized as an effective intervention for improving mobility, function, and quality of life in patients with advanced knee pathology. 1 In 2010, nearly 686,000 TKA procedures were performed in the United States. 2 This number has since surpassed 1 million annually and is projected to reach approximately 1.26 million procedures by 2030. 3

According to the Centers for Disease Control and Prevention, approximately 14% of U.S. adults—approximately 35.5 million people—are affected by chronic kidney disease (CKD). 4 Advances in medical management have improved survival among patients with CKD. However, this has been accompanied by a rising burden of CKD-related complications. Renal osteodystrophy (ROD), a complex disorder of bone metabolism, develops in nearly all individuals with CKD over the course of their disease, affecting both adult and pediatric populations.

ROD, a bone metabolism disorder secondary to CKD, is marked by disruption of mineral and hormonal homeostasis. Impaired renal phosphate excretion leads to hyperphosphatemia, while diminished activation of vitamin D causes hypocalcemia, stimulating secondary hyperparathyroidism (SHPT) with elevated parathyroid hormone (PTH) levels. This drives bone resorption and demineralization, further exacerbated by fibroblast growth factor 23 (FGF23)-mediated suppression of vitamin D. The resulting disturbances contribute to abnormal bone turnover, fractures, skeletal deformities, cardiovascular events, and increased mortality. ROD occurs most often in advanced CKD stages (3–5) and affects the majority of patients with progressive renal impairment. Although biochemical abnormalities such as elevated PTH and FGF23 may appear early in CKD, overt bone pathology and clinical sequelae strongly correlate with declining glomerular filtration rate (GFR). 5 6

Despite the increasing number of TKA procedures performed in patients with ROD, the impact of this condition on postoperative outcomes remains poorly defined. This knowledge gap underscores the need for a comprehensive evaluation to guide clinical decision-making. Accordingly, we conducted a national cohort study to assess the influence of ROD on TKA outcomes, examining patient demographics, comorbidity burden using the Charlson Comorbidity Index (CCI), length of hospital stay (LOS), hospital charges, and a broad range of medical and surgical complications. Clarifying these associations is essential for optimizing perioperative management, improving patient outcomes, and informing health care resource planning in this vulnerable and expanding patient population.

## Materials and Methods

### Data Source

The National (Nationwide) Inpatient Sample (NIS), a key component of the Healthcare Cost and Utilization Project (HCUP), is the largest publicly available database for inpatient care in the United States, encompassing information on more than 7 million hospitalizations. This extensive resource captures discharge data from a stratified 20% sample of U.S. hospitals and offers comprehensive insights across all payer categories. The sampling method captures 97% of all hospital discharges nationwide. The large sample size of the NIS makes it ideal for generating both national and regional estimates, and it is particularly useful for studying rare pathologies, uncommon treatments, and specific demographic groups. Data from the NIS include total hospital charges, patient demographics, LOS, insurance type, and diagnostic and procedural codes from the International Classification of Diseases, both Ninth and Tenth Revisions (ICD-9-CM, ICD-10-CM).

### Data Collection

**Fig. 1:**
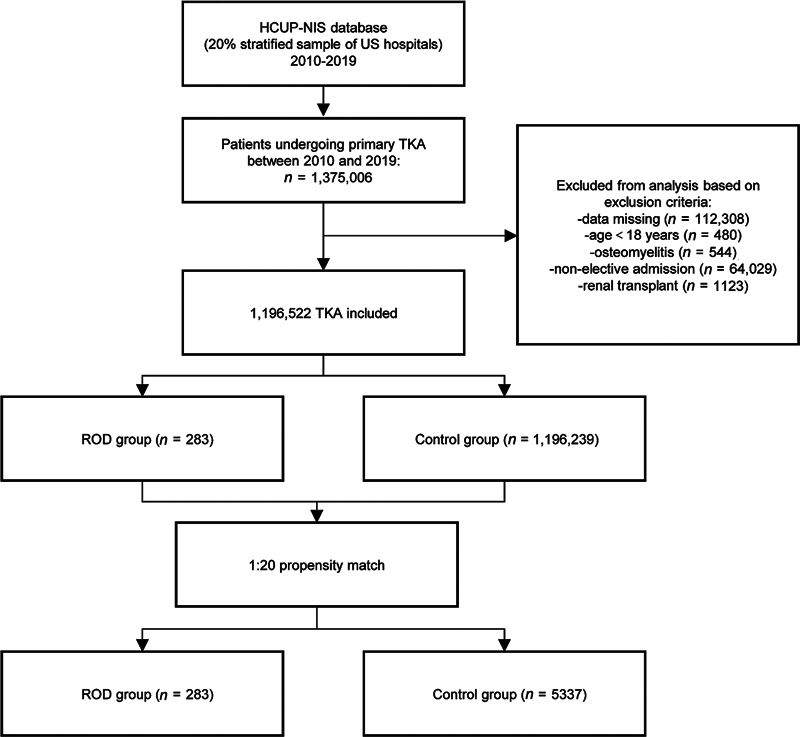
Inclusion and exclusion criteria as well as the analysis flowchart. HCUP-NIS, Healthcare Cost and Utilization Project-National (Nationwide) Inpatient Sample; ROD, renal osteodystrophy; TKA, total knee arthroplasty.

The study was exempted from the Institutional Review Board (IRB) review due to the use of de-identified and publicly accessible data. In the NIS database from 2010 to 2019, we identified patients according to ICD-9-CM and ICD-10-CM procedural codes of TKA (ICD-9-CM 81.54 and ICD-10-PCS codes: 0SRC07Z, 0SRC0J9, 0SRC0JA, 0SRC0JZ, 0SRC0KZ, 0SRC0L9, 0SRC0LA, 0SRC0LZ, 0SRD07Z, 0SRD0J9, 0SRD0JA, 0SRD0JZ, 0SRD0KZ, 0SRD0L9, 0SRD0LA, 0SRD0LZ) (see Supplementary material , available in the online version). We identified 1,375,006 patients undergoing primary TKA between 2010 and 2019. Exclusion criteria were applied sequentially: Patients with missing data ( *n* = 112,308), age <18 years ( *n* = 480), osteomyelitis ( *n* = 544), non-elective admissions ( *n* = 64,029), or prior renal transplantation ( *n* = 1,123) were excluded. A final cohort of 1,196,522 adults undergoing elective TKA was analyzed. Patients were subsequently divided into two groups: Those with ROD (ICD-9-CM 588.0 and ICD-10-CM N25) and those without ROD ( Fig. 1 ). Of this cohort, 283 patients (0.02%) were categorized into the ROD group, while the remaining 1,196,239 patients (99.98%) comprised the control group. After 1:20 PSM, the ROD group ( *n* = 283) was successfully matched with 5,337 control patients.

Medical complications such as sepsis, hemorrhagic anemia, thrombocytopenia, convulsion, acute myocardial infarction (AMI), cardiac arrest, heart failure, chest pain, arrhythmia, peripheral vascular disease, deep vein thrombosis (DVT), acute cerebrovascular disease, stroke, delirium, respiratory failure, pneumonia, pulmonary embolism (PE), gastrointestinal bleeding, acute renal failure (ARF), urinary retention, urinary tract infection (UTI), transfusion, and shock were included. 7 Surgical complications such as hematoma, wound disruption, wound infection, periprosthetic fracture, periprosthetic joint dislocation, periprosthetic joint infection, and lower limb peripheral nerve injuries were included. 8 The CCI score was used to stratify patients (CCI = 0, 1, 2, ≥3). 8 Patient demographics, hospital characteristics, comorbidities (including CCI and CKD stage), medical and surgical complications, and outcomes were defined as shown in Table 1 .

**Table 1:** Variable used in binary logistic regression analysis

Variables categories	Specific variables
Patient demographics	Age (≤64 and ≥65 years), sex (male and female), race (White, Black, and Other)
Hospital characteristics	Teaching status of hospital (non-teaching, teaching), location of hospital (rural, urban)
Comorbidities	Diabetes (uncomplicated), diabetes (with chronic complications), obesity, dialysis, Charlson Comorbidity Index, CKD stage
Medical complications	Sepsis, hemorrhagic anemia, thrombocytopenia, convulsion, AMI, cardiac arrest, heart failure, chest pain, arrhythmia, peripheral vascular disease, DVT, acute cerebrovascular disease, stroke, delirium, respiratory failure, pneumonia, PE, GI bleeding, ARF, urinary retention, UTI, transfusion, shock
Surgical complications	Hematoma, wound disruption, wound infection, periprosthetic fracture, periprosthetic joint dislocation, periprosthetic joint infection, lower limb peripheral nerve injuries
Outcome	Patient disposition, length of stay, total charges

Abbreviations: AIDS, acquired immunodeficiency syndrome; AMI, acute myocardial infarction; ARF, acute renal failure; CKD, chronic kidney disease; DVT, deep vein thrombosis; PE, pulmonary embolism; UTI, urinary tract infection.

### Statistical Analysis

All statistical analyses were performed using SPSS version 27.0 (IBM Corp., Armonk, NY). A 1:20 PSM algorithm was applied, accounting for preoperative factors including age group (18–65, ≥65), sex, race (White, Black, Other), obesity, tobacco use, diabetes (without complications), diabetes (with complications), CCI category (0, 1, 2, ≥3), and CKD stage. Descriptive statistics were used to compile demographic and clinical characteristics. Analyses were performed on both the unmatched cohort and the propensity-matched cohort. Continuous variables are presented as median (interquartile range, IQR); comparisons between groups were made using the Mann–Whitney U test as the data distribution was non-normal. Categorical variables are presented as frequency (percentage); comparisons were made using chi-square tests or Fisher's exact test (for counts <5). 9 Statistical significance was defined as a two-sided *p* -value of <0.05. 10 Multivariate logistic regression analyses were performed to calculate odds ratio (OR) and 95% confidence interval (CI) for postoperative complications, comparing the ROD group to the control group. To adjust for potential residual confounding, dialysis status and the propensity score itself were included as covariates in the multivariate logistic regression models. 11 Multivariate logistic regression using the stepwise method was performed to identify the independent association of ROD with postoperative complications and outcomes.

## Results

### Patient Demographics

Baseline characteristics of the two groups were evaluated and compared. Prior to propensity matching, the median age in the ROD group was 68 years (IQR: 62–74), compared with 67 years (IQR: 60–73) in the control group ( *p* = 0.009). The ROD group comprised 41.7% males (118/283) and 58.3% females (165/283), while the control group included 38.1% males (455,703/1,196,239) and 61.9% females (740,536/1,196,239) ( *p* = 0.212). Significant racial disparities were observed: The ROD group had lower proportions of White patients (68.2% vs. 82.5%) but higher proportions of Black (19.8% vs. 8.0%) and Other racial groups' patients (12.0% vs. 9.6%) compared with controls ( *p* < 0.001). Obesity was more prevalent in the ROD group (38.5% vs. 26.9%, *p* < 0.001), as was diabetes with complications (24.4% vs. 3.7%, *p* < 0.001). Patients with CCI ≥3 were substantially overrepresented in the ROD group (96.5% vs. 82.1%, *p* < 0.001), while CKD stage distribution differed significantly between groups ( *p* < 0.001). Following 1:20 PSM for age, sex, race, obesity, tobacco use, diabetes (with/without complications), CCI category, and CKD stage, all demographic variables showed no significant differences between the matched ROD ( *n* = 283) and control groups ( *n* = 5,337; Table 2 and Supplementary Material S2 [available in the online version only]).

**Table 2:** Variables in propensity matching

	Before matching			After matching		
	ROD group ( *n* = 283)	Control group ( *n* = 1,196,239)	*p* -Value	ROD group ( *n* = 283)	Control group ( *n* = 5,337)	*p* -Value
Age (years)	68 (62–74)	67 (60–73)	0.009	68 (62–74)	68 (62–75)	0.084
Age group						
18–65	93 (32.9%)	497,927 (41.6%)	0.003	93 (32.9%)	1,585 (29.7%)	0.799
≥ 65	190 (67.1%)	698,312 (58.4%)		190 (67.1%)	3,752 (70.3%)	
Gender						
Male	118 (41.7%)	455,703 (38.1%)	0.212	118 (41.7%)	2,080 (39.0%)	0.360
Female	165 (58.3%)	740,536 (61.9%)		165 (58.3%)	3,257 (61.0%)	
Race						
White	193 (68.2%)	986,479 (82.5%)	<0.001	193 (68.2%)	3,774 (70.7%)	0.632
Black	56 (19.8%)	95,379 (8.0%)		56 (19.8%)	997 (18.7%)	
Other	34 (12.0%)	114,381 (9.6%)		34 (12.0%)	566 (10.6%)	
Obesity	109 (38.5%)	322,869 (26.9%)	<0.001	109 (38.5%)	2,007 (37.6%)	0.758
Tobacco used	49 (17.3%)	170,391 (14.2%)	0.139	49 (17.3%)	966 (18.1%)	0.738
Diabetes						
Diabetes (without complications)	40 (14.1%)	218,300 (18.3%)	0.073	40 (14.1%)	797 (14.9%)	0.713
Diabetes (with complications)	69 (24.4%)	44,374 (3.7%)	<0.001	69 (24.4%)	1,249 (23.4%)	0.705
CCI						
0	0 (0%)	4,095 (0.3%)	<0.001	0 (0%)	0 (0%)	0.980
1	1 (0.4%)	36,588 (3.1%)		1 (0.4%)	20 (0.4%)	
2	9 (3.2%)	173,830 (14.5%)		9 (3.2%)	181 (3.4%)	
≥ 3	273 (96.5%)	981,726 (82.1%)		273 (96.5%)	5,136 (96.2%)	
CKD stage						
No CKD/data missing	130 (45.9%)	1,127,601 (94.2%)	<0.001	130 (45.9%)	2,677 (50.2%)	0.001
I	1 (0.4%)	921 (0.1%)		1 (0.4%)	28 (0.5%)	
II	4 (1.4%)	6,649 (0.6%)		4 (1.4%)	196 (3.7%)	
III	46 (16.3%)	34,295 (2.9%)		46 (16.3%)	977 (18.3%)	
IV	24 (8.5%)	3,304 (0.3%)		24 (8.5%)	461 (8.6%)	
V	68 (24.0%)	1,665 (0.1%)		68 (24.0%)	68 (1.27%)	
CKD unspecified stage	10 (3.5%)	22,344 (1.9%)		10 (3.5%)	10 (3.5%)	

Abbreviations: CCI, Charlson Comorbidity Index; CI, confidence interval; CKD, chronic kidney disease; OR, odds ratio; ROD, renal osteodystrophy.

### Patient Admission Characteristics

Before matching, ROD patients were more frequently treated in teaching hospitals (61.1% vs. 54.3%, *p* = 0.002) compared with controls, but no significant difference was observed in urban hospital utilization (87.3% vs. 89.8%, *p* = 0.171). Both before and after matching, the ROD group showed significantly longer median LOS (3 vs. 3 days before matching, *p* < 0.001; 3 vs. 3 days after matching, *p* < 0.001) and substantially higher median total charges ($58,550 vs. $50,192 before matching, *p* < 0.001; $58,550 vs. $18,004 after matching, *p* < 0.001; Table 3 ).

**Table 3:** Hospital characteristics and outcomes before and after 1:20 matching

	Before matching			After matching		
	ROD group ( *n* = 283)	Control group ( *n* = 1,196,239)	*p* -Value	ROD group ( *n* = 283)	Control group ( *n* = 5,337)	*p* -Value
Teaching hospital	173 (61.1%)	648,916 (54.3%)	0.002	173 (61.1%)	3,077 (57.7%)	0.248
Urban hospital	247 (87.3%)	1,073,583 (89.8%)	0.171	247 (87.3%)	3,354 (59.3%)	0.779
LOS (median, d)	3 (2–4)	3 (2–3)	<0.001	3 (2–4)	3 (2–3)	<0.001
TOTCHG (median, $)	58,550 (42,381–83,506)	50,192 (36,684–70,704)	<0.001	58,550 (42,381–83,506)	18,004 (11,601–36,303)	<0.001

Abbreviations: LOS, length of stay; ROD, renal osteodystrophy; TOTCHG, total charge.

### Unmatched Complications and Adverse Events during Hospital Admission

Compared with the control group, ROD patients had significantly higher rates of complications, including sepsis (OR: 7.80, 95% CI: 1.94–31.40), hemorrhagic anemia (OR: 1.35, 95% CI: 1.20–1.77), thrombocytopenia (OR: 4.85, 95% CI: 3.05–7.73), convulsion (OR: 6.99, 95% CI: 2.88–16.93), cardiac arrest (OR: 8.65, 95% CI: 1.21–61.77), heart failure (OR: 5.99, 95% CI: 4.11–8.76), chest pain (OR: 3.21, 95% CI: 1.20–8.61), peripheral vascular disease (OR: 2.47, 95% CI: 1.44–4.22), acute cerebrovascular disease (OR: 6.06, 95% CI: 2.99–12.24), stroke (OR: 6.84, 95% CI: 3.52–13.29), delirium (OR: 2.92, 95% CI: 1.21–7.71), respiratory failure (OR: 5.28, 95% CI: 2.18–12.79), pneumonia (OR: 6.99, 95% CI: 3.30–14.81), ARF (OR: 6.43, 95% CI: 4.43–9.34), transfusion (OR: 2.11, 95% CI: 1.49–2.99), shock (OR: 22.32, 95% CI: 3.12–159.84), wound infection (OR: 5.76, 95% CI: 1.43–23.16), periprosthetic fracture (OR: 9.70, 95% CI: 3.16–26.06), periprosthetic joint dislocation (OR: 4.89, 95% CI: 2.02–11.85), and lower limb peripheral nerve injuries (OR: 2.95, 95% CI: 1.66–5.26). Conversely, no significant differences were observed for AMI, arrhythmia, DVT, PE, gastrointestinal bleeding, urinary retention, UTI, hematoma, wound disruption, or periprosthetic joint infection ( Table 4 ).

**Table 4:** Relationship between renal osteodystrophy and postoperative complications before matching

Complications	Univariate analysis			Multivariate logistic regression		
	ROD	Control	*p* -Value	OR	95% CI	*p* -Value
Medical complications						
Sepsis	2 (0.71%)	1,086 (0.09%)	0.028	7.80	1.94–31.40	**0.004**
Hemorrhagic anemia	68 (24.03%)	227,761 (19.04%)	0.033	1.35	1.20–1.77	**0.033**
Thrombocytopenia	19 (6.71%)	17,489 (1.46%)	<0.001	4.85	3.05–7.73	**<0.001**
Convulsion	5 (1.77%)	3,072 (0.26%)	<0.001	6.99	2.88–16.93	**<0.001**
Acute myocardial infarction	1 (0.35%)	4,099 (0.34%)	0.621	1.03	0.15–7.35	0.975
Cardiac arrest	1 (0.35%)	490 (0.04%)	0.11	8.65	1.21–61.77	**0.031**
Heart failure	30 (10.6%)	23,200 (1.94%)	<0.001	5.99	4.11–8.76	**<0.001**
Chest pain	4 (1.41%)	5,320 (0.44%)	0.039	3.21	1.20–8.61	**0.021**
Arrhythmia	0 (0%)	9,305 (0.78%)	0.29	–	–	–
Peripheral vascular disease	14 (4.95%)	24,721 (2.07%)	<0.001	2.47	1.44–4.22	**<0.001**
Deep vein thrombosis	2 (0.71%)	3,478 (0.29%)	0.199	2.44	0.61–9.81	0.209
Acute cerebrovascular disease	8 (2.83%)	5,718 (0.48%)	<0.001	6.06	2.99–12.24	**<0.001**
Stroke	9 (3.18%)	5,717 (0.48%)	<0.001	6.84	3.52–13.29	**<0.001**
Delirium	5 (1.77%)	7,320 (0.61%)	0.031	2.92	1.21–7.71	**0.018**
Respiratory failure	5 (1.77%)	4,063 (0.34%)	0.003	5.28	2.18–12.79	**<0.001**
Pneumonia	7 (2.47%)	4,323 (0.36%)	<0.001	6.99	3.30–14.81	**<0.001**
Pulmonary embolism	2 (0.71%)	3,561 (0.30%)	0.207	2.38	0.59–9.58	0.221
Gastrointestinal bleeding	0 (0%)	717 (0.06%)	1	–	–	–
Acute renal failure	31 (10.95%)	22,456 (1.88%)	<0.001	6.43	4.43–9.34	**<0.001**
Urinary retention	5 (1.77%)	26,629 (2.23%)	0.601	0.79	0.37–1.91	0.601
Urinary tract infection	8 (2.83%)	19,738 (1.65%)	0.151	1.73	0.86–3.50	0.125
Surgical complications						
Transfusion	36 (12.72%)	77,259 (6.46%)	<0.001	2.11	1.49–2.99	**<0.001**
Hematoma	2 (0.71%)	3,656 (0.31%)	0.215	2.32	0.58–9.33	0.235
Shock	1 (0.35%)	190 (0.02%)	0.044	22.32	3.12–159.84	**0.002**
Wound disruption	0 (0%)	1,016 (0.08%)	1	–	–	–
Wound infection	2 (0.71%)	1,477 (0.12%)	0.049	5.76	1.43–23.16	**0.014**
Periprosthetic fracture	4 (1.41%)	1,765 (0.15%)	0.001	9.70	3.16–26.06	**<0.001**
Periprosthetic joint dislocation	5 (1.77%)	4,384 (0.37%)	0.004	4.89	2.02–11.85	**<0.001**
Periprosthetic joint infection	2 (0.71%)	4,628 (0.39%)	0.299	1.83	0.46–7.37	0.393
Lower limb peripheral nerve injuries	12 (4.24%)	17,681 (1.48%)	0.001	2.95	1.66–5.26	**<0.001**

Abbreviations: CI, confidence interval; OR, odds ratio; ROD, renal osteodystrophy.

### Matched Complications and Adverse Events during Hospital Analysis

Using a 1:20 PSM algorithm, 283 patients with ROD were matched to 5,337 control patients. After matching, ROD patients demonstrated significantly higher rates of several medical and surgical complications, including thrombocytopenia (OR: 3.17, 95% CI: 1.91–5.24), convulsion (OR: 6.92, 95% CI: 2.45–19.56), heart failure (OR: 2.28, 95% CI: 1.52–3.41), chest pain (OR: 3.40, 95% CI: 1.16–9.97), acute cerebrovascular disease (OR: 3.00, 95% CI: 1.40–6.41), stroke (OR: 3.29, 95% CI: 1.60–6.77), pneumonia (OR: 3.86, 95% CI: 1.66–8.96), ARF (OR: 2.33, 95% CI: 1.57–3.45), periprosthetic fracture (OR: 7.07, 95% CI: 2.18–22.93), periprosthetic joint dislocation (OR: 4.63, 95% CI: 1.74–12.34), and lower limb peripheral nerve injuries (OR: 2.53, 95% CI: 1.37–4.69). In contrast, no statistically significant differences were observed between groups for complications such as sepsis, hemorrhagic anemia, cardiac arrest, AMI, arrhythmia, peripheral vascular disease, DVT, delirium, respiratory failure, PE, gastrointestinal bleeding, urinary retention, UTI, transfusion, shock, hematoma, wound disruption, wound infection, or periprosthetic joint infection ( Table 5 ).

**Table 5:** Relationship between renal osteodystrophy and postoperative complications after 1:20 matching

Complications	Univariate analysis			Multivariate logistic regression		
	ROD	Control	*p* -Value	OR	95% CI	*p* -Value
Medical complications						
Sepsis	2 (0.7%)	12 (0.2%)	0.141	1.21	0.28–5.24	0.798
Hemorrhagic anemia	68 (24.0%)	1,175 (20.8%)	0.187	1.16	0.88–1.55	0.282
Thrombocytopenia	19 (6.7%)	107 (1.9%)	<0.001	3.17	1.91–5.24	**<** **0.001**
Convulsion	5 (1.8%)	19 (0.3%)	0.005	6.92	2.45–19.56	**<** **0.001**
Acute myocardial infarction	1 (0.4%)	22 (0.4%)	1	0.59	0.08–4.35	0.602
Cardiac arrest	1 (0.4%)	5 (0.1%)	0.254	1.58	0.20–12.60	0.665
Heart failure	30 (10.6%)	143 (2.5%)	<0.001	2.28	1.52–3.41	**<0.001**
Chest pain	4 (1.4%)	25 (0.4%)	0.047	3.40	1.16–9.97	**0.026**
Arrhythmia	0 (0%)	59 (1%)	0.116	NA	NA	0.994
Peripheral vascular disease	14 (4.9%)	155 (2.7%)	0.029	1.18	0.67–2.05	0.570
Deep vein thrombosis	2 (0.7%)	10 (0.2%)	0.109	1.91	0.44–8.27	0.388
Acute cerebrovascular disease	8 (2.8%)	33 (0.6%)	<0.001	3.00	1.40–6.41	0.005
Stroke	9 (3.2%)	33 (0.6%)	<0.001	3.29	1.60–6.77	**0.001**
Delirium	5 (1.8%)	46 (0.8%)	0.094	1.84	0.72–4.68	0.199
Respiratory failure	5 (1.8%)	34 (0.6%)	0.036	2.19	0.27–17.47	0.459
Pneumonia	7 (2.5%)	28 (0.5%)	<0.001	3.86	1.66–8.96	**0.002**
Pulmonary embolism	2 (0.7%)	19 (0.3%)	0.264	NA	NA	0.965
Gastrointestinal bleeding	0 (0%)	7 (0.1%)	1	NA	NA	0.998
Acute renal failure	31 (11%)	208 (3.7%)	<0.001	2.33	1.57–3.45	**<** **0.001**
Urinary retention	5 (1.8%)	142 (2.5%)	0.433	0.74	0.30–1.83	0.515
Urinary tract infection	8 (2.8%)	101 (1.8%)	0.202	1.09	0.53–2.24	0.821
Transfusion	36 (12.7%)	368 (6.5%)	<0.001	1.23	0.85–1.78	0.276
Shock	1 (0.4%)	0 (0%)	0.048	6.61	0.57–77.03	0.132
Surgical complications						
Hematoma	2 (0.7%)	17 (0.3%)	0.228	1.30	0.30–5.62	0.726
Wound disruption	0 (0%)	9 (0.2%)	1	NA	NA	0.998
Wound infection	2 (0.7%)	9 (0.2%)	0.094	NA	NA	0.966
Periprosthetic fracture	4 (1.4%)	13 (0.2%)	0.007	7.07	2.18–22.93	**0.001**
Periprosthetic joint dislocation	5 (1.8%)	26 (0.5%)	0.015	4.63	1.74–12.34	**0.002**
Periprosthetic joint infection	2 (0.7%)	42 (0.7%)	1	0.81	0.19–3.39	0.777
Lower limb peripheral nerve injuries	12 (4.2%)	83 (1.5%)	0.002	2.528	1.37–4.69	**0.003**

Abbreviations: CI, confidence interval; OR, odds ratio; ROD, renal osteodystrophy.

Excluding advanced CKD led to substantially larger effect estimates for several postoperative complications: Thrombocytopenia (OR: 9.25, 95% CI: 3.74–22.88), convulsion (20.74, 5.13–83.85), pneumonia (27.66, 6.13–124.87), respiratory failure (40.89, 3.69–453.86), PE (20.44, 2.86–146.23), DVT (13.62, 2.26–82.22), ARF (8.39, 3.20–21.98), and UTI (5.47, 2.90–10.32; all *p* ≤ 0.004; Supplementary Material S3 [available in the online version only]). By contrast, associations for acute cerebrovascular disease and lower limb peripheral nerve injury were attenuated and lost significance in Supplementary Material S3 (available in the online version only). Many estimates in Supplementary Material S3 (available in the online version only) are imprecise (wide 95% CIs), reflecting smaller event counts after exclusion; therefore, the amplified ORs should be interpreted cautiously.

## Discussion

With the increasing prevalence of CKD, the number of patients with ROD undergoing TKA is expected to rise. 5 In our national-level cohort analysis using the NIS database, we found that ROD was independently associated with an increased risk of multiple postoperative complications even after rigorous PSM.

The ROD group was older than the control group, aligning with previous research by Morina, 12 which reported that ROD predominantly affects individuals aged between 60 and 69. This may reflect cumulative mineral metabolism disturbances that worsen with CKD progression. Our study also showed that patients with ROD experienced significantly longer hospital stays and incurred higher total charges, consistent with prior findings in patients with end-stage renal disease (ESRD) undergoing TKA, where prolonged LOS and elevated costs were also reported.

Before PSM, ROD patients had higher rates of a wide range of complications. However, many of these differences became non-significant after matching for key variables including CKD stage, comorbidities, and demographics—highlighting the importance of properly adjusting for confounding. Notably, even after adjustment, ROD patients continued to exhibit higher rates of several major complications.

Specifically, the ROD group had significantly increased odds of thrombocytopenia, which is consistent with studies indicating that patients with moderate-to-severe CKD are predisposed to platelet dysfunction and reduced platelet count, elevating bleeding risk. 13 14 Similarly, the markedly increased rate of convulsions in ROD patients may relate to the accumulation of uremic toxins like methylguanidine, which are known to provoke seizures and cognitive disturbances. 15

We also found significantly higher rates of heart failure and chest pain in the ROD group. Beyond the known burden of CKD itself, ROD—as the skeletal manifestation of chronic kidney disease–mineral and bone disorder (CKD-MBD)—also contributes independently to elevated heart failure and chest pain rates. First, ROD fosters vascular calcification and arterial stiffness via dysregulated mineral handling (especially hyperphosphatemia, hypocalcemia, and SHPT), prompting osteoblastic transformation of vascular smooth muscle cells and decreased bone turnover to fuel medial calcification. This stiffening increases left ventricular afterload, leading to hypertrophy and ultimately congestive heart failure. 16 17 Mechanistically, the bone–vascular axis in ROD essentially shifts calcium and phosphate balance away from bone toward vascular deposition, exacerbating cardiac workload even independent of GFR. 18 Skeletal complications like rib osteosclerosis may also produce chest discomfort from bone pain, as noted in ROD patients with metabolic bone disease visible on radiographs. 19

ROD confers additional cerebrovascular vulnerability through mechanisms distinct from general CKD effects. ROD, driven by aberrant mineral homeostasis (notably hyperphosphatemia, SHPT, and disordered bone turnover), is intimately linked to arterial medial and intimal calcification, which extends into the intracranial arterial circulation. 20 This calcification increases arterial stiffness and pulse pressure, impairing cerebral autoregulation and elevating ischemic and hemorrhagic stroke risk. CKD-MBD has been recognized as a key contributor to stroke risk beyond traditional vascular risk factors. While end-stage kidney disease remains a major risk factor, abnormalities in bone metabolism and vascular calcification independently elevate cerebrovascular event rates. 21 22

The significantly increased rate of pneumonia in patients with ROD may reflect pathophysiologic alterations beyond kidney dysfunction alone. ROD, as a component of CKD–MBD, is characterized by dysregulated bone turnover, which can compromise immune defense mechanisms. SHPT—a hallmark of ROD—has been shown to impair both innate and adaptive immunity. Elevated PTH levels can modulate T-cell activation, reduce neutrophil chemotaxis, and alter macrophage function, predisposing to increased susceptibility to infections, including pneumonia. Animal and clinical studies demonstrate that PTH excess contributes to lymphoid tissue atrophy and impaired cytokine signaling. 23

Surgical complications such as periprosthetic fracture, joint dislocation, and lower limb peripheral nerve injuries were also more common in ROD patients. Bone fragility in ROD stems from disrupted bone remodeling due to SHPT, impaired vitamin D metabolism, and elevated FGF23 levels. 24 25 These factors weaken skeletal integrity and neuromuscular function, making patients more prone to mechanical instability and injury.

In a sensitivity analysis excluding patients with CKD stages 3–5 ( Supplementary Material S3 [available in the online version only]), the associations between ROD and postoperative complications were largely consistent with those observed in the primary analysis and, for several outcomes, became substantially stronger. Specifically, after removing patients with advanced baseline renal impairment, markedly higher odds were observed for thrombocytopenia, convulsion, pneumonia, respiratory failure, PE, DVT, ARF, and UTI. Notably, several of these associations, which were non-significant or modest in the primary model, became large and statistically significant in Supplementary Material S3 (available in the online version only). These amplified estimates likely reflect both true differences in risk among patients without advanced CKD and instability related to smaller event counts following exclusion. Indeed, the wide 95% CIs around many Supplementary Material S3 (available in the online version only) point estimates suggest sparse data effects, underscoring the need for cautious interpretation. Supplementary Material S3 (available in the online version only) complements the primary findings by demonstrating that the association between ROD and major postoperative complications persists—and in some cases intensifies—among patients without advanced CKD, while emphasizing the importance of considering data precision and sample size in sensitivity analyses.

This study has several limitations, which are common in database studies. Confounding variables not included in the database cannot be controlled, and there is potential for misclassification bias, particularly for patients with ROD, due to the absence of laboratory data and detailed medical records in the NIS. Additionally, the study was limited to examining postoperative complications, as postoperative functional outcomes and long-term survivorship data were not available. Because the NIS relies on hospital discharge diagnosis codes, some patients coded with ROD may not have been separately coded for CKD if treating clinicians considered that information already encompassed by the ROD designation. This coding practice likely underestimates the proportion of CKD among ROD patients and represents a limitation of administrative databases. Nevertheless, by including CKD stage as a covariate in our PSM, we have minimized residual confounding due to underlying renal dysfunction. Therefore, our findings—demonstrating an independent association between ROD and postoperative complications after TKA—remain robust and clinically informative.

## Conclusion

In this large, propensity-matched national cohort of primary TKA patients, ROD emerged as an independent predictor of markedly worse perioperative outcomes. Despite equivalent baseline demographics and comorbidity burden, ROD patients incurred higher hospital charges and experienced longer inpatient stays. Critically, they sustained significantly greater odds of both medical complications—thrombocytopenia, convulsions, heart failure, chest pain, acute cerebrovascular events, stroke, respiratory failure, pneumonia, and ARF—and surgical complications, including periprosthetic fractures, joint dislocations, and lower limb peripheral nerve injuries. These findings underscore the profound impact of disordered mineral and bone metabolism—beyond renal dysfunction alone—on skeletal integrity, cardiovascular and cerebrovascular resilience, immune competence, and neuromuscular stability. Recognition of ROD as a risk factor for a broad spectrum of adverse events should prompt heightened preoperative assessment, multidisciplinary perioperative planning, and targeted resource allocation to mitigate complications in this vulnerable population. Ultimately, tailoring optimization strategies for bone and mineral disorders may improve safety, reduce costs, and enhance functional recovery in ROD patients undergoing TKA.
