# Supplementary material for: Renal Osteodystrophy as a Risk Factor for Postoperative Complications after Knee Arthroplasty: A National In-Patient Sample Study
Source: J Knee Surg. 2025 Dec 30;39(7):355–63. doi: 10.1055/a-2756-0149 (PMC13288436; doi:10.1055/a-2756-0149)
Supplement: Supplementary file 1 — Supplementary Material [file 10-1055-a-2756-0149-s24dec0254oa-1.pdf]

Table S1 ICD codes used for analysis

Diagnosis/Procedure	ICD-9 Codes	ICD-10 Codes
**CKD Stage 1**	5851	N181
**CKD Stage 2**	5852	N182
**CKD Stage 3**	5853	N183
**CKD Stage 4**	5854	N184
**CKD Stage 5**	5855	N185
**CKD ESRD**	5856	N186
**ROD**	5880	N250
**Osteomyelitis**	730X; 730X0-9; 7301X0-9; 7302X0-9	M8600-M8609; M8610-M8619; M862-M866; M868X0-9; M869
**Acute Renal Failure**	5845; 5846; 5847; 5848; 5849	N170; N171; N172; N178; N179
**Myocardial Infarction**	41041-41043; 41051-41053; 41061-41063; 41071-41073; 41081-41083; 41091-41093	I2101; I2102; I2109; I21111; I2119; I2121; I2129; I213; I214; I219; I21A1; I21A9; I220-I229
**Blood Loss Anemia**	2851	D62
**Pneumonia**	4800-4809; 481; 4820-4829; 4830-4838; 4841-4848; 485; 486; 4870; 5070-5078; 99731-99732	J120-J129; J13-J159; J160-J189; J678-J679; J954
**Blood Transfusion**	99.XX (e.g., 9903;	30233N1

© Thieme. All rights reserved.

Diagnosis/Procedure	ICD-9 Codes	ICD-10 Codes
	9904; 9905; 9907)	
**Pulmonary Embolism**	41511; 41513; 41519	I2602; I2609; I2692; I2699
**Deep Vein Thrombosis (DVT)**	45340-45342; 4536; 45381-45389; 4539; 4511-4519	I824XX; I825XX
**Stroke**	430-4329; 43300-43391; 43400-43491	I6000-I609; I610-I619; I6200-I629; I6300-I639
**Urinary Tract Infection**	5901-5909; 5950-5959; 5970-5978; 5990; 9975	N390; N3000-N3091; N340-N343; T83021A/T83021D/T83021S
**Sepsis**	99591-99592	A400-A419
**Fever**	78060; 78062	R5082; R509
**Periprosthetic Fracture**	99644	T84010A-T84019A; M9665-M96679; M9701XA-M9712XA
**Periprosthetic Dislocation**	99642	T84020A-T84029A
**Periprosthetic Infection**	99666	T8450XA-T8459XA
**Wound Infection**	9985; 6826; 6829; 8900-8902; 8940-8942	T814XXA/T814XXD/T814XXS
**Seroma/Hematoma**	99811-99813; 72992; 71915-71916	L7621-L7622; L7631-L7634; M7981
**Injury to Peripheral**	956X	S840-S849X

© Thieme. All rights reserved.

Diagnosis/Procedure	ICD-9 Codes	ICD-10 Codes
**Nerve**		
**Delirium**	2930-2939; 29281; 78097	F05; R4181-R4189; F060; F062
**Chest Pain**	78650-78659	R072; R0781-R0789; R079
**Arrhythmia**	30230H; 30230J; 30230N; 30253N; 4260-42689	I470; I498; I499
**Wound Dehiscence**	9983	T8130XA-T8132XA
**Gastrointestinal Bleeding**	5780-5789	K91841; K91871; K91873; K250-K286; K922
**Urinary Retention**	788.20-788.29	R33.8; R33.9
**TKA**	8154	0SRC07Z; 0SRC0J9-Z; 0SRC0KZ; 0SRC0L9-Z; 0SRD07Z; 0SRD0J9-Z; 0SRD0KZ; 0SRD0L9-Z
**Transfusion**	9900-9908; V582	30230H1-30263S0

© Thieme. All rights reserved.
