# Supplementary material for: Renal Osteodystrophy as a Risk Factor for Postoperative Complications after Knee Arthroplasty: A National In-Patient Sample Study
Source: J Knee Surg. 2025 Dec 30;39(7):355–63. doi: 10.1055/a-2756-0149 (PMC13288436; doi:10.1055/a-2756-0149)
Supplement: Supplementary file 2 — Supplementary Material [file 10-1055-a-2756-0149-s24dec0254oa-2.pdf]

Table S1. Balance Summary from Matching Output

Variable	Treated Mean	Control Mean	Std. Mean Diff.	Var. Ratio	eCDF Mean Diff.	eCDF Max Diff.	Std. Pair Dist.
distance	0.0009	0.0002	0.5504	5.8194	0.2337	0.4646	0.0003
FEMALE	0.5830	0.6191	-0.0730		0.0360	0.0360	0.1075
Age_Group	1.6714	1.5838	0.1862	0.9112	0.0438	0.0876	0.1207
Tobacoo	0.1731	0.1424	0.0812		0.0307	0.0307	0.0604
NEW_RACE	1.4382	1.2710	0.2393	1.2557	0.0557	0.1427	0.0965
CM_DM	0.1413	0.1825	-0.1182		0.0412	0.0412	0.0839
CM_DMCX	0.2438	0.0371	0.4814		0.2067	0.2067	0.1039
CM_OBESE	0.3852	0.2699	0.2368		0.1153	0.1153	0.0678
CCI_Group	2.9611	2.7832	0.8425	0.1773	0.0445	0.1440	0.0009
CKD_Stage	2.3781	0.2840	0.8468	3.4120	0.2920	0.4828	0.0793

 © Thieme. All rights reserved.

Table S2. Standardized Mean Differences After Matching

Variable	Type	Standardized Mean Difference (Adj.)
distance	Distance	0.0001
FEMALE	Binary	-0.0206
Age_Group_2	Binary	-0.0103
Tobacoo	Binary	-0.0102
NEW_RACE	Continuous	-0.0136
CM_DM	Binary	-0.0029
CM_DMCX	Binary	-0.0339
CM_OBESE	Binary	-0.0011
CCI_Group	Continuous	0.0008
CKD_Stage	Continuous	0.0632

 © Thieme. All rights reserved.
