# Supplementary material for: Renal Osteodystrophy as a Risk Factor for Postoperative Complications after Knee Arthroplasty: A National In-Patient Sample Study
Source: J Knee Surg. 2025 Dec 30;39(7):355–63. doi: 10.1055/a-2756-0149 (PMC13288436; doi:10.1055/a-2756-0149)
Supplement: Supplementary file 3 — Supplementary Material [file 10-1055-a-2756-0149-s24dec0254oa-3.pdf]

Thieme

SM3 Multivariate Logistic regression after PSM (the control group excluded patients with CKD stages 3-5 and those with unknown CKD stages)

Complications	Multivariate Logistic Regression		
	**OR**	**95% CI**	**P**
**Medical complications**			
Sepsis	NA	NA	NA
hemorrhagic anemia	1.326	0.853–2.060	0.210
thrombocytopenia	9.246	3.737–22.875	**<0.001**
convulsion	20.738	5.129–83.850	**<0.001**
acute myocardial infarction	NA	NA	NA
cardiac arrest	20.293	1.262–326.215	**0.034**
heart failure	3.575	1.573–8.125	**0.002**
chest pain	5.829	1.199–28.332	**0.029**
arrhythmia	NA	NA	NA
peripheral vascular disease	1.357	0.539–3.412	0.517
deep vein thrombosis	13.621	2.257–82.215	**0.004**
acute cerebrovascular disease	2.553	0.759–8.588	0.130
stroke	3.431	1.173–10.032	**0.024**
delirium	4.076	0.884–18.789	0.072
respiratory failure	40.894	3.685–453.859	**0.003**
pneumonia	27.662	6.13–124.87	**<0.001**
pulmonary embolism	20.439	2.857–146.233	**0.003**
gastrointestinal bleeding	NA	NA	NA
acute renal failure	8.391	3.202–21.984	**<0.001**
urinary retention	1.265	0.39–4.12	0.696
urinary tract infection	5.471	2.901–10.319	**<0.001**
transfusion	0.852	0.392–1.854	0.687
shock	NA	NA	NA
**Surgical complications**			
hematoma	NA	NA	NA
wound disruption	NA	NA	NA
wound infection	NA	NA	NA
periprosthetic fracture	10.212	1.854–56.257	**0.008**
periprosthetic joint dislocation	4.733	1.333–16.814	**0.016**
periprosthetic joint infection	NA	NA	NA
lower limb peripheral nerve injuries	2.277	0.798–6.493	0.124
